# The use of cardiac specific biomarkers for cardiovascular risk assessment in asymptomatic individuals: is it a valuable approach in clinical practice?

**DOI:** 10.1016/j.ijcrp.2021.200099

**Published:** 2021-09-24

**Authors:** Speranza Rubattu

**Affiliations:** Department of Clinical and Molecular Medicine, School of Medicine and Psychology, Sapienza University of Rome, IRCCS Neuromed, Pozzilli, Italy

Cardiovascular diseases (CVDs) are a consequence of genetic and environmental factors and they carry a heavy socio-economic burden. Appropriate preventive strategies are needed to limit the development and consequences of CVDs, particularly ischemic heart diseases. As discussed in recent guidelines and consensus documents [1,2], a currently debated issue is the detection of asymptomatic individuals at increased CVD risk. For this purpose, more effective diagnostic strategies to precisely identify the level of risk are required.

Circulating biomarkers may represent suitable tools to identify and stratify an individual's risk and design specific preventive approaches. Circulating biomarkers are tipically molecules belonging to different biological systems and mechanisms regulating the functions of cells, tissues and organs. As such, biomarkers are tightly connected with either physiological or pathological conditions, including those of the cardiovascular system. Furthermore, their circulating levels can be measured with appropriate and very sensitive assays to reflect normality or disease.

The detection of fine changes of biomarkers levels may anticipate the transition from a healthy to a disease condition in asymptomatic individuals. The latter application fits perfectly with one of the major tasks of primary CVD prevention, that is to detect individuals at risk. Furthermore, this approach has been already tested in relation to the two widely studied cardiac biomarkers, the amino-terminal brain natriuretic peptide (NT-proBNP) and cardiac troponin I (cTnI).

NT-proBNP, synthetized by ventricular cardiac myocytes in response to hemodynamic stimuli, belongs to the natriuretic peptides family and is involved in the regulation of volume, electrolytes balance and blood pressure level. Based on its functional roles, we can consider it mainly as a marker of cardiac stress. In addition to its relevant diagnostic and prognostic roles, NT-proBNP level has been reported to be associated with CVDs occurrence both in the Framingham USA population and in several European cohorts [3,4]. A remarkable result was achieved few years ago through one of the largest epidemiological study, involving 40 cohorts of the general population from all over the world (>95000 subjects), demonstrating the ability of NT-proBNP plasma level to significantly predict heart failure, stroke, and ischemic heart disease, and to improve the cardiovascular risk stratification when added to C-reactive protein and HDL-cholesterol levels [5].

Levels of cTnI, particularly when measured with a very sensitive assay, can be slightly higher than normal in apparently healthy subjects as a consequence of the physiological turnover of cardiomyocytes. Further increases of cTnI level, which are above the cut-off value of 10 ng/L for women and 12 ng/L for men, respectively, and are well below the 99 % percentile URL to exclude myocardial infarction, indicate the presence of a subtle myocardial tissue damage. A meta-analysis involving 28 cohorts (>154000 subjects) showed that individuals carrying the highest hs-cTn level had a 43 % increased risk for cardiovascular events and a 67 % increased risk for fatal CVD [6]. Therefore, cTnI values that are elevated, but below the 99th percentile value, are able to predict mortality and cardiovascular risk.

The abovementioned results confirm our expectations based on the known pathophysiological implications of NT-proBNP and cTnI. Of note, increased levels of these cardio-specific biomarkers, provide complementary pathophysiological and clinical information, and may indicate greater cardiovascular risk than that identified with only one elevated biomarker.

Although these studies indicate the suitability of both NT-proBNP and cTnI levels for CVD risk prediction, recent international guidelines on cardiovascular prevention do not include the use of biomarkers to guide treatment. Indeed, the use of biomarkers as a potentially useful tool to identify asymptomatic individuals at increased cardiovascular risk remains an open issue. In fact, the preliminary results from the measurement of both NT-proBNP and cTnI levels, although very promising, do not fulfil the rigorous criteria needed for a biomarker to be approved for CVD prevention, as stated by the task force of the European Society of Cardiology [7].

The lack of a favourable analytical profile and the high degree of biological variability within the population have so far represented major limitations. In this regard, NT-proBNP levels appear more problematic due the higher degree of intra-individual variability.

In order to definitively demonstrate the true value of cardio-specific biomarkers in CVD prevention, more studies should be performed, including studies in multiple ethnic groups, and more attention should be paid to subgroups (e.g. by sex, age and duration of follow up). These studies should strengthen the role of both NT-proBNP and cTnI levels in CVD risk prediction in apparently healthy individuals. Furthermore, they should provide more evidence about the ability of the cardiac biomarkers to improve the current risk-estimation models that are based on the conventional risk factors. We also need more robust demonstration of their ability to reflect, through a reduction of their plasma level, the impact of intervention strategies, including pharmacological intervention, aimed at modifying the CVD risk. Most importantly, the cost-benefit ratio, including serial measurements, should be clearly assessed.

Once the cardio-specific biomarkers fulfil this rigorous assessment, the question about their additive value in CVD risk prediction can be determined. Their measurement into the routine clinical practice could become a reality with the aim of improving CVDs prevention in the general population.
